# Gluten-Free Diet in Children: An Approach to a Nutritionally Adequate and Balanced Diet

**DOI:** 10.3390/nu5114553

**Published:** 2013-11-18

**Authors:** Francesca Penagini, Dario Dilillo, Fabio Meneghin, Chiara Mameli, Valentina Fabiano, Gian Vincenzo Zuccotti

**Affiliations:** Department of Pediatrics, University of Milan, Luigi Sacco Hospital, Milan 20157, Italy; E-Mails: dilillo.dario@hsacco.it (D.D.); meneghin.fabio@hsacco.it (F.M.); mameli.chiara@hsacco.it (C.M.); fabiano.valentina@hsacco.it (V.F.); gianvincenzo.zuccotti@unimi.it (G.V.Z.)

**Keywords:** celiac disease, gluten-free diet, children, nutritional complications, balanced diet

## Abstract

Gluten-free diet (GFD) is the cornerstone treatment for celiac disease (CD). GFD implies a strict and lifelong elimination from the diet of gluten, the storage protein found in wheat, barley, rye and hybrids of these grains, such as kamut and triticale. The absence of gluten in natural and processed foods, despite being the key aspect of GFD, may lead to nutritional consequences, such as deficits and imbalances. The nutritional adequacy of GFD is particularly important in children, this the age being of maximal energy and nutrient requirements for growth, development and activity. In recent years, attention has focused on the nutritional quality of gluten-free products (GFPs) available in the market. It is well recognized that GFPs are considered of lower quality and poorer nutritional value compared to the gluten-containing counterparts. The present review focuses on the nutritional adequacy of GFD at the pediatric age, with the aim being to increase awareness of the potential complications associated with this diet, to identify strategies in order to avoid them and to promote a healthier diet and lifestyle in children with CD.

## 1. Introduction

Celiac disease (CD) is a chronic systemic autoimmune disorder caused by a permanent intolerance to gluten proteins in genetically susceptible individuals. Gluten is a general term used to describe a mixture of storage proteins, including prolamins, hordeins and secalins found in wheat, barley and rye, respectively. These proteins may exert a toxic effect on intestinal mucosa in genetically susceptible individuals by triggering an immune-mediated response, responsible for the typical villous atrophy and lymphocyte infiltrate in small intestine mucosa seen in CD. In fact, these proteins contain epitopes that undergo deamidation, an important process for the binding of the CD associated human leukocyte antigen (HLA) DQ2/DQ8 haplotypes with T-lymphocytes, activating an autoimmune response [1,2,3].

A lifelong strict gluten-free diet (GFD) is the only available treatment for CD. Adherence to GFD leads to regression of symptoms, normalization of histological and laboratory findings and reduces the risk of CD associated complications [4]. Within the range of gluten-free foods, a distinction must be made between those that are naturally gluten-free and those that are made gluten-free through a process of purification. There are several foods that are naturally gluten-free, such as rice, corn, potatoes and a number of different grains, seeds and legumes. Historically, rice, corn and potatoes have been the first natural substitutes for gluten-containing grains. Today, a number of different grains, including pseudo-cereals, offer increased variety, improved palatability to GFD and are a good source of carbohydrates, protein, dietary fiber, vitamins and polyunsaturated fatty acids [5,6]. The commercially available gluten-free (GF) products are processed foods purified of gluten. The elimination of this storage protein inevitably alters the macro- and micro-nutrient composition, thus the nutritional value. First, wheat is not only a major source of protein, but also of iron, folates and B vitamins (thiamin, riboflavin and niacin); in fact, GF products are often low in these nutrients, as opposed to their gluten containing equivalents [7,8,9]. The various gluten-free and gluten containing foods are listed in Table 1.

**Table 1:** Gluten-free and gluten containing cereals and other foods.

Gluten-Free Cereals	Gluten Containing Cereals
**Cereals and minor cereals**	Wheat Barley Rye Kamut Malt Triticale
Corn	
Rice	
Sorghum	
Oats *	
Teff	
Millet	
**Pseudo-cereals**	
Amaranth	
Quinoa	
Buckwheat	
**Vegetable foods**	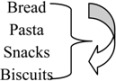
Vegetables	
Fruits	
Nuts	
**Other plant foods**	All foods produced with any of the gluten containing cereals mentioned above. Numerous foods available in supermarkets and grocery stores, including meat products, sweets and beer, contain gluten.
Potatoes	
Tapioca	
Soybean	
Vegetable oils	
**Animal foods**	
Dairy products	
Egg	
Meat	
Fish	

* Controversial; see paragraph on oats.

### 1.1. Macronutrients

Studies show that gluten-free products (GFPs) often have a greater carbohydrate and lipid content than their gluten containing equivalents [10,11,12,13]. Segura *et al.* analyzed the nutritional composition of a range of GF breads and found that these are starchy-based foods with a high glycemic index (estimated between 83.3 and 96.1 *vs.* 71 for white wheat flour bread), with low protein and high fat content [9]. With regards to lipid content and composition, Caponio *et al.* showed that commercially available GF biscuits are richer in saturated fatty acids compared to the gluten containing equivalents [13]. All these characteristics have a negative effect on health, and this should be seriously taken into account, because the limited choice of food products in the diet of children with CD induces a high consumption of packaged GFPs, such as snacks and biscuits.

### 1.2. Micronutrients and Minerals

It has been shown that some commercially available GFPs have a lower content of folates, iron and B vitamins or are not consistently enriched/fortified compared to their gluten containing counterparts [7,8,9]. Thompson [7,8] conducted two studies on US commercially available GFPs. The author analyzed the folate, iron and B vitamins (thiamin, riboflavin and niacin) composition of these products and compared them with the composition of their gluten containing counterparts, finding that GFPs were significantly lower in folates, iron and B vitamins.

### 1.3. Dietary Fiber

Some studies have reported that GFD is associated with a lower intake of dietary fiber than a standard gluten containing diet [14,15]. A study conducted in the USA on adults showed that the diet of CD patients on GFD was low in fiber intake [15]. This phenomenon is likely to be related to the composition of many GF foods made with starches and/or refined flours with low content in fiber. In fact, during the refining process, the outer layer of grain containing most of the fiber is removed, leaving only the starchy inner layer.

## 2. Nutritional Imbalances in Children with Celiac Disease Following a GFD

Studies conducted in adults and children show that approximately 20%–38% of patients with CD have nutritional complications, such as calorie/protein imbalance, dietary fiber, mineral and vitamin deficiencies [16,17,18,19]. These complications may be encountered both at diagnosis and during follow-up, whilst on GFD [9,18]. At diagnosis, the deficiencies are often secondary to nutrient malabsorption due to mucosal damage. Studies show that the more pronounced the villous atrophy, the greater the nutritional deficiencies, with lower levels of iron, copper, folate, vitamin B-12 and zinc [19]. For CD patients on GFD, the nutritional complications are likely to be caused by the poor nutritional quality of the GFPs mentioned above and by the incorrect alimentary choices of CD patients. The most common nutritional deficiencies encountered in adults with CD, at diagnosis and during GFD, are described in Table 2.

**Table 2:** Common nutrient deficiencies in adults with celiac disease (CD) at diagnosis and after Gluten-free diet (GFD). Modified from Cynthia Kupper [20].

Common Nutrient Deficiencies in Subjects with Celiac Disease			
At Diagnosis	GFD	GFD Products	Long-Term GFD
Calorie/protein			
Fiber	Fiber	Fiber	Fiber
Iron	Iron	Iron	
Calcium	Calcium		
Vitamin D	Vitamin D		
Magnesium	Magnesium		
Zinc			
Folate, niacin, vitamin B12	Folate, niacin, vitamin B12	Folate, niacin, vitamin B12	Folate, niacin, vitamin B12
Riboflavin	Riboflavin	Riboflavin	Riboflavin

Numerous studies focus their attention specifically on the nutrient intakes of CD children and adolescents on GFD. Mariani *et al.* [21] studied the nutritional habits of 47 adolescents (aged 10–20 years) affected by CD, by means of a three-day alimentary diary. A comparison with the Italian and American recommended daily allowance (RDAs) was done for the intakes of macronutrients, fiber, iron and calcium. The results showed that CD subjects followed a high-protein and high-lipid diet with low intakes of carbohydrates, iron, calcium and fiber compared to the recommended daily intakes. Furthermore, Hopman *et al.* [22] studied the nutrient intake of 37 adolescents with CD (aged 13–16 years) following a strict GFD and compared the intakes with a control group in the same age category. In the CD group, the intake of saturated fat was significantly higher than recommended by both the American and Dutch RDAs; the intake of fiber and iron was significantly lower than recommended. Furthermore, the comparison between the two groups showed that the intake of fiber and iron was lower in the CD group compared to controls (*p* *<* 0.05). öhlund *et al.* [23] conducted a study in 2010 on 30 children aged 4–17 years with CD and on GFD, using a five-day food record. High intakes of saturated fat and sucrose and low intakes of dietary fiber, vitamin D and magnesium compared to recommendations (New Nordic Nutrition Recommendations, 2004) was observed.

An elegantly conducted study by Zuccotti *et al.* compared the dietary intake of CD children on GFD to a group of healthy children (healthy controls, HC) and evaluated the contribution of commercially available GFPs on the observed nutritional intake [24]. The study showed that the daily intake of vitamin D was significantly lower in the CD compared to the HC group (vitamin D median value 3.1 μg + 0.6 *vs.* 3.1 µg + 2.8, *p* *<* 0.01). With regards to macronutrients, the intake of simple sugars, fats and protein exceeded the national recommendations for health in both the CD and HC groups. The total daily energy intake was significantly higher in the CD group compared to HCs (8961.8 KJ day^−1^ *vs.* 5761.0 KJ day^−1^; *p* *<* 0.001). In the CD group, the carbohydrate-derived energy was higher, while the lipid-derived energy was lower, compared to the HC group. Protein-derived energy did not differ between the two groups. In the CD group, the contribution of commercially available GFPs on daily energy intake was studied. The main finding was that these products provided 36.3% of the total daily energy intake (3253.1 KJ day^−1^ out of 8961.8 KJ day^−1^) in these patients. Analyzing the contribution of the macronutrients derived from GFPs on the total daily energy intake, it was observed that protein derived from GFPs accounted for 7.3% of the total energy derived from protein, representing 18% of total daily energy intake. With regards to lipids, GFPs contributed a median of 12.9 g day^−1^ of the median total daily fat intake of 73.0 g, which is equivalent to 17.7% of energy from fat. Furthermore, Mariani *et al.* [21] conducted a nutritional analysis of children with CD. The author found that children complying with a strict GFD had significantly greater nutritional imbalance in their diet than did children cheating on their GFD. More troubling, the incidence of children who were overweight or obese was more frequent (72%) in the strict GFD group compared with the children not following a strict GFD (51%) and healthy age-matched controls (47%). A study conducted by Ferrara *et al.* [25] compared the caloric intake and fat consumption of 50 children with CD following a GFD with 50 healthy children. A significant increase in fat consumption was observed in children with CD compared to healthy children (72.5 + 37.2 g *vs.* 52.9 + 35.4 g per 100 g of food, *p* *<* 0.008). Furthermore, a significant difference in fat intake was observed between the two groups (10.21 + 3.15 g per 100 g of food in CD group *vs.* 7.46 + 2.91 g/100 g in control group, *p* *=* 0.004).

## 3. Effects of Gluten-Free Diet on Anthropometric Parameters

The scientific literature on anthropometric parameters in children with CD on GFD provide contrasting data. On the one hand, there is evidence that good compliance with GFD is associated with a positive effect on anthropometric parameters, including: the reduction of fat and the recovery of lean body mass [26], normalization of body mass index (BMI) in both previously underweight and overweight subjects [27] and acceleration of linear growth [28]. A study conducted in children with CD and obesity at diagnosis showed a significant reduction in BMI after 12 months of GFD [29]. Another study conducted by Brambilla *et al.* found a lower frequency of being overweight and of obesity in children with CD, both at diagnosis and during GFD, compared to healthy controls [30]. Even though the frequency of being overweight and of obesity in the CD group increased on GFD, it remained lower than observed in the general population.

On the other hand, there are also studies that suggest that GFD may have a negative effect on body composition and anthropometric parameters in subjects with CD [31]. Mariani *et al.* [21] first reported the high prevalence of being overweight and of obesity in CD adolescents on GFD; the authors found that more than 50% of CD adolescents were overweight during GFD. However, in the latter paper, the authors used a relative body weight >110%, rather than BMI, to define being overweight, probably leading to an overestimation of being overweight. Furthermore, a study conducted by Valletta *et al.* showed that the frequency of being overweight in children with CD was nearly doubled after one year of GFD [32]. Potential explanations for the undesirable weight gain and obesity observed in these studies are possible overfeeding as the intestinal mucosa heals, consumption of less complex carbohydrates and fiber and more sugars, proteins and saturated fats in GFD. The conflicting data may in part be caused by differences in the timing of anthropometric assessment. Many children with CD, after introduction of GFD, may initially gain excessive weight and only thereafter start to show catch-up growth and normalization of weight. Even though the effect of GFD on body weight and BMI remains a controversial issue, it remains fundamental for pediatricians to be aware of the possible nutritional consequences of GFD for which early recognition can be crucial in the prevention of obesity-related complications.

## 4. Dietary Advices for a Nutritionally Adequate and Balanced Diet in Children with Celiac Disease

### 4.1. Education and Compliance

The first step towards a balanced diet starts from early education on CD and GFD, possibly provided by a skilled dietitian and/or by a physician with expert knowledge in CD. The diet is complicated and can be overwhelming if not presented using a thorough and proactive approach. Early education is fundamental to promote adherence to GFD. In fact, studies focusing on compliance to GFD indicate that adherence is compromised by a number of factors, including a lack of education and continued support by a physician and dietitian [33,34]. In a study conducted by Charalampopoulos *et al.* on determinants of the adherence of GFD in children with CD, baseline education was one of the main determinant factors influencing compliance, suggesting the importance for frequent reinforcement and an accurate explanation of dietary recommendations [34].

### 4.2. Dietary Intake

In CD children on GFD, the recommended distribution of daily calorie intake for a healthy and balanced diet does not differ from that recommended to the general population. According to the dietary reference intake values (DRI), the total daily dietary calories should be ideally obtained as 55% from complex and simple carbohydrates, 15% from dietary protein and 25%–30% or less from lipids. The intake of unsaturated fat (monounsaturated and polyunsaturated) should be preferred. Monounsaturated and polyunsaturated fatty acids should provide more than 15% and 10% of total calories, respectively. They are found in foods, such as vegetable oils, nuts, seeds and fish high in lipid content, such as salmon, trout and herring. Thus, it is important to inform and recommend children with CD on the ideal distribution of the daily calorie intake.

### 4.3. Gluten-Free Foods

It is advisable to prefer consumption of naturally GF foods, since it has been shown that they are more balanced and complete under both the macro- and micro-nutrient point of view. In fact, these foods are considered to have a higher nutritional value in terms of energy provision, lipid composition and vitamin content as opposed to the commercially purified GF products. Within the range of naturally GF foods, it is preferable to consume those rich in iron and folic acid, such as leafy vegetables, legumes, fish and meat. During explanation of naturally GF foods to patients, it is a good approach for healthcare professionals to bear in mind the local food habits and recipes of each country. This may provide tailored dietary advice, improving acceptance and compliance to GFD. Furthermore, increasing awareness on the availability of the local naturally GF foods may help promote their consumption, resulting in a more balanced and economically advantageous diet. Indeed, these aspects should always be addressed during dietary counseling. With regards to the commercially purified GFPs, it is recommended to pay special attention to the labeling and chemical composition. In European countries, the currently accepted definition of GF is the one designed by Codex Alimentarius (the gluten-free certification organization, 2007) [35]. The term “gluten-free” refers only to foods containing less than 20 ppm of gluten. In addition, the claim “very low gluten” is used for foods, such as bread, produced using cereals that have been specially processed to remove most of the gluten and containing less than 30 mg daily. Furthermore, some GFPs are enriched/fortified with vitamins and/or minerals, thus the choice of these products is preferred, to prevent the deficiencies associated with GFD, mentioned above. Clear labeling of GFPs and education of CD patients on how to interpret them is fundamental to helping CD subjects make safer and more informed food choices.

### 4.4. Pseudo-Cereals and Minor-Cereals

Pseudo-cereals, such as amaranth, quinoa and buckwheat, and other minor cereals represent a healthy alternative to frequently used ingredients in gluten-free products. They are a good source of carbohydrates, protein, dietary fiber, vitamins and polyunsaturated fatty acids [36]. In fact, the fiber content in these grains ranges from seven to 10 g/100 g [37], which is higher compared to those of other plant foods and cereals and approximately the same as the content in wheat (fiber 9.5 g/100 g). Furthermore, they are a valid source of protein, as their content is superior to that of wheat in terms of the quantity and quality of proteins: in particular, lysine, arginine, histidine, methionine and cysteine can be found in high amounts [38,39]. Although the lipid content of pseudo-cereals is higher compared to other plant foods, they are characterized by a higher content of unsaturated fatty acids, in particular, α-linolenic acid [40,41], beneficial for the prevention of cardiovascular diseases. In addition, higher concentrations of folic acid have been found in quinoa (78.1 μg/100 g) and amaranth (102 μg/100 g) with respect to wheat (40 μg/100 g). Furthermore, both amaranth and quinoa are also good sources of riboflavin, vitamin C and vitamin E [42]. In addition, pseudo-cereals permit a wider variety of foods, broadening the choice for CD children when selecting foods. Under the economic point of view, these grains offer a less expensive alternative with respect to standard gluten-free choices; also, this aspect could help increase dietary compliance by reducing the economic burden of the diet [43]. The nutritional advantages of pseudo-cereals are listed in Table 3.

**Table 3:** Nutritional advantages of pseudo-cereals (amaranth, buckwheat and quinoa).

Nutritional Characteristics of Amaranth, Buckwheat and Quinoa
High fiber content, 7–10 g/100 g, approximately the same as wheat fiber 9.5 g/100 g.
High protein content, 10.9%–15.2% of dry mass *vs.* 11.7% of dry mass in wheat.
High quality amino acids: lysine, arginine, histidine, methionine and cysteine.
Source of unsaturated fatty acids, in particular, α-linolenic acid.
High content of folic acid: quinoa and amaranth, 78.1 µg/100 g and 102 µg/100 g, respectively, *vs.* 40 µg/100 g in wheat.
Source of vitamins: B2, B6, riboflavin, vitamin C and E.
Source of minerals: the content is twice as high as in other cereals.

### 4.5. Oats

The inclusion of oats in the GFD has been, for many years, and still is a matter of debate, because it was thought that avenin (the storage protein found in oats) was also toxic to CD patients. Moreover, attention has been focused on the issue of the frequent cross-contamination of oats with gluten-containing grains. Studies have demonstrated that when consumed in moderation, oats free from cross-contamination are well tolerated by most children [44,45]. Under the nutritional point of view, oats represent a good source of iron, dietary fiber, thiamin and zinc and, in addition, have a good palatability [46]. A study conducted by Størsund *et al.* in CD children suggests that oats may improve the nutritional value of GFD and, in view of the good palatability, may also help increase compliance [47]. Recently, Lee *et al.* demonstrated that adding three servings of gluten-free alternative grains, including oats, positively impacts the nutrient profile (fiber, thiamin, riboflavin, niacin, folate and iron) of the grain portion of the gluten-free diet [48].

### 4.6. Vitamins and Minerals

Meat, fish, fruit and vegetables are an important natural source of vitamins, minerals and trace elements. In view of the possible micronutrient deficiencies associated with GFPs, an appropriate consumption of these foods should be advised in children with CD. In particular, fruit and vegetables are low in energy and rich in vitamins and minerals; moreover, they contain phytochemicals and antioxidant compounds that exert a protective effect against diseases associated with oxidative damage [49]. The intake of at least five portions of fruit and vegetables a day should be recommended in children with CD. Minerals (calcium, phosphorus, sodium, potassium, chloride and magnesium) and trace elements (iron, zinc and selenium) are also contained in a significant amount in pseudo-cereals, in which the content can be twice as high as in other cereals. For example, in teff, iron and calcium contents (11–33 mg/100 g and 100–150 mg/100 g, respectively) are higher than those of wheat, barley, sorghum and rice.

### 4.7. Nutritional Follow-Up

Continuous long-term follow-up is crucial to promote adherence to GFD and for early identification of nutritional deficiencies and/or metabolic imbalances. Ideally, a skilled dietitian with knowledge in CD and GFD should be an integral part of the healthcare team. A child’s nutritional status should be accurately assessed at diagnosis and at each follow-up, which ideally should be performed at six months post commencement of GFD and then annually, post-diagnosis. The evaluation of nutritional status should start from a thorough and accurate dietary history and include the assessment of anthropometric parameters (weight, height and body mass index). Adherence to GFD should be assessed, and information on how to safely broaden food choices and interpret food labeling should be given. Early identification and correction of nutritional deficiencies should be regularly addressed. Table 4 summarizes the key points of nutritional follow-up in children with CD.

**Table 4:** Recommended timing for nutritional follow-up. BMI: body mass index.

Nutritional Follow-Up	
When?	How?
Diagnosis 6 months post commencement of GFD Annually post diagnosis	Accurate dietary history
	Evaluation of nutritional status
	Anthropometric parameters, (weight, height, BMI)
	Physical examination (attention to signs of malnutrition)

**Figure 1:**
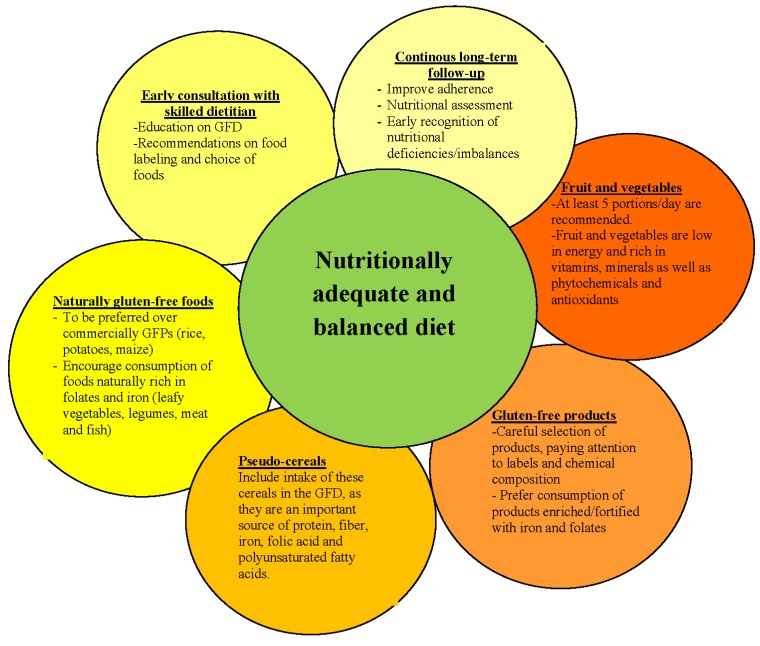
Schematic representation of proposed approach to a nutritionally adequate and balanced gluten-free diet.

## 5. Conclusions

Gluten-free diet, the only available treatment for CD, if not carried out with attention, may paradoxically lead to nutritional imbalances, which should be avoided, particularly at the pediatric age, the phase of maximal growth and development. Increasing awareness on the possible nutritional deficiencies associated with GFD may help healthcare professionals and families tackle the issue by starting from early education on GFD and clear dietary advice on how to choose the most appropriate gluten-free foods. Figure 1 summarizes, by means of a schematic representation, a proposed approach towards a nutritionally adequate and balanced gluten-free diet. Further studies on the technological and nutritional properties of the alternative cereals as wheat replacements are needed to confirm their role in improving the intake of protein, iron, calcium and fiber and reducing nutritional deficiencies in children with CD. Their role in the economic burden of the diet and their effect on compliance should also be further investigated. Furthermore, a promising field for gluten-free diet is food biotechnologies. By means of this science, it would be worth considering genetically modifying the amino acid sequence of gluten storage proteins, in order to make them free of those domains high in prolines and glutamines, which are responsible for the toxicity.
